# Breaking the Taboo: Four Cases of Thoracic Segmental Spinal Anesthesia in Modern Surgical Practice

**DOI:** 10.7759/cureus.115280

**Published:** 2026-08-27

**Authors:** Paolo Scimia, Massimiliano Luca D'Agostino, Antonio De Cato, Franco Marinangeli, Luca Gentili

**Affiliations:** 1 Department of Anesthesia, Intensive Care Unit, Giuseppe Mazzini Hospital, Teramo, ITA; 2 Department of Clinical Medicine, Public Health, Life, and Environmental Sciences, University of L’Aquila, L'Aquila, ITA; 3 Department of Anesthesia, Intensive Care Unit, Santa Maria Goretti Hospital, Latina, ITA

**Keywords:** high-risk patients, laparoscopic surgery, regional anesthesia, spontaneous ventilation, thoracic spinal anesthesia

## Abstract

Thoracic segmental spinal anesthesia (TSSA) is an old neuraxial technique that has recently regained attention as a possible alternative to general anesthesia (GA) in selected surgical patients. Its segmental nature, low-dose local anesthetic requirement, and ability to preserve spontaneous ventilation may be particularly relevant in high-risk patients in whom airway manipulation, endotracheal intubation, positive-pressure ventilation, and systemic anesthetic exposure could represent an additional physiological burden. We report four selected high-risk patients undergoing procedures from different surgical specialties under TSSA: emergency videolaparoscopic adnexal detorsion in early pregnancy; retrograde intrarenal surgery (RIRS) in a patient with arthrogryposis multiplex congenita and restrictive ventilatory impairment; laparoscopic appendectomy in a patient with severe chronic obstructive pulmonary disease (COPD) and chronic cor pulmonale; and laparoscopic cholecystectomy in a morbidly obese patient with obstructive sleep apnea. In all cases, TSSA was tailored according to patient characteristics and surgical requirements through individualized selection of puncture level, local anesthetic baricity, adjuvants, sedation strategy, and respiratory support. All procedures were completed without conversion to GA, airway rescue, clinically relevant respiratory deterioration, or postoperative neurological complications. Quality of recovery was high in all patients. These cases suggest that TSSA may represent a versatile, physiology-preserving anesthetic strategy in carefully selected high-risk patients, provided that it is performed by experienced practitioners within a multidisciplinary plan and with a clear backup strategy.

## Introduction

Thoracic segmental spinal anesthesia (TSSA) is a neuraxial technique in which low doses of local anesthetic are injected at thoracic interspaces to obtain a selective sensory and sympathetic block over the metameres involved in surgery. Although historically less commonly used than lumbar spinal anesthesia, TSSA has recently attracted renewed interest as a potential alternative to general anesthesia (GA) in selected patients and surgical settings [1,2]. Recent reviews have described its application across different surgical domains, including abdominal, laparoscopic, thoracic, breast, obstetric, orthopedic, and urologic procedures [3].

The renewed interest in TSSA reflects a broader shift toward physiology-preserving anesthetic strategies, especially in patients in whom the risks of airway manipulation, endotracheal intubation, positive-pressure ventilation, or systemic anesthetic exposure may be clinically relevant. However, TSSA should not be interpreted as a simple technical variation of conventional lumbar spinal anesthesia. It is better understood as a complex anesthetic strategy requiring careful patient selection, individualized dosing, knowledge of thoracic neuraxial anatomy, advanced monitoring, shared decision-making with the surgical team, and a clear backup strategy [4].

Historically, the clinical feasibility of segmental thoracic spinal anesthesia was highlighted by van Zundert et al. in a patient with severe lung disease undergoing laparoscopic cholecystectomy [5]. Subsequent anatomical and clinical studies supported the rationale for cautious thoracic-level puncture and low-dose segmental blockade [6,7]. Nevertheless, concerns regarding technical difficulty, spinal cord injury, hemodynamic instability, and management of patient comfort during laparoscopic surgery continue to limit broader adoption.

We report four cases in which TSSA was used in selected high-risk patients undergoing procedures belonging to different surgical specialties. The aim of this case series is not to suggest that TSSA should replace GA, but to illustrate how it may be integrated into a tailored perioperative strategy when the expected burden of GA may be disproportionate to patient-specific risks.

## Case presentation

Each patient was adequately informed of the risks and benefits of the proposed anesthesiological management. Written informed consent was obtained from all patients, and the anesthetic plan was shared with the surgical team and, when appropriate, with family members during the preoperative evaluation.

The common anesthetic objective was to provide an adequate segmental block for surgery while preserving spontaneous ventilation. The level of puncture, local anesthetic baricity, adjuvants, sedation regimen, and respiratory support were individualized according to the patient's comorbidities and surgical requirements. The main clinical and anesthetic features of the four cases are summarized in Table 1. Additional quantitative perioperative data, including American Society of Anesthesiologists (ASA) physical status, procedure duration, block onset time, maximal sensory level, analgesic requirement, intraoperative hemodynamic and respiratory ranges, and post-discharge follow-up, are reported in Table 2. The Quality of Recovery-15 (QoR-15), a validated postoperative recovery questionnaire, was administered before hospital discharge in all patients [8].

**Table 1:** Main clinical and anesthetic features of the four cases BMI: body mass index; COPD: chronic obstructive pulmonary disease; CPAP: continuous positive airway pressure; IV: intravenous; OSAS: obstructive sleep apnea syndrome; PEEP: positive end-expiratory pressure; POD: postoperative day; QoR-15: Quality of Recovery-15; RASS: Richmond Agitation-Sedation Scale; RIRS: retrograde intrarenal surgery; TSSA: thoracic segmental spinal anesthesia

Case	Patient	Main risk factors	Procedure	Surgical field	Neuraxial technique	Intraoperative management	Analgesic requirement	Outcome
1	29-year-old pregnant woman	• Moderate obesity; • First-trimester pregnancy	Videolaparoscopic adnexal detorsion	Gynecology	• Puncture level: T10-T11; • Dose and concentration: 0.25% hyperbaric bupivacaine 5 mg + 0.25% isobaric ropivacaine 11.25 mg; • Adjuvants: No	• Propofol 1 mg/kg/h; • RASS: -1; • Spontaneous breathing; • No vasopressor requirement	• 2 g IV paracetamol	• Complications: No • Bromage: 0 • POD: 2 • QoR-15: 142
2	46-year-old woman	• Arthrogryposis multiplex congenita; • Rotoscoliosis; • Restrictive ventilatory impairment	RIRS for renal stones	Urology	• Puncture level: T11-T12; • Dose and concentration: 0.25% isobaric levobupivacaine 10 mg + 0.25% hypobaric levobupivacaine 5 mg; • Adjuvants: Dexmedetomidine 4 µg	• Propofol 1 mg/kg/h; • RASS: -1; • Spontaneous breathing; • No vasopressor requirement	• 1 g IV paracetamol • 30 mg IV ketorolac	• Complications: No • Bromage: 0 • POD: 3 • QoR-15: 144
3	78-year-old woman	• Severe COPD; • Chronic cor pulmonale; • Long-term oxygen therapy	Laparoscopic appendectomy	General surgery	• Puncture level: T9-T10; • Dose and concentration: 0.25% hyperbaric bupivacaine 5 mg + 0.25% isobaric ropivacaine 11.25 mg; • Adjuvants: Morphine 50 µg	• Propofol 1 mg/kg/h; • RASS: -1; • Spontaneous breathing; • No vasopressor requirement	• No rescue analgesia	• Complications: No • Bromage: 0 • POD: 2 • QoR-15: 148
4	52-year-old man	• Severe obesity; • OSAS on CPAP; • Hypertension; • Type 2 diabetes	Laparoscopic cholecystectomy	General surgery	• Puncture level: T11-T12; • Dose and concentration: 0.25% hypobaric ropivacaine 5 mg + 0.25% isobaric ropivacaine 10 mg; • Adjuvants: No	• Dexmedetomidine 0.4 µg/kg/h; • RASS: -1; • Home CPAP (PEEP 8 cmH₂O); • No vasopressor requirement	• No rescue analgesia	• Complications: No • Bromage: 0 • POD: 2 • QoR-15: 145

**Table 2:** Quantitative perioperative data and post-discharge follow-up ASA: American Society of Anesthesiologists physical status; bpm: beats per minute; EtCO₂: end-tidal carbon dioxide; h: hours; HR: heart rate; MAP: mean arterial pressure; min: minutes; mmHg: millimeters of mercury; POD: postoperative day; SpO₂: peripheral oxygen saturation; C: cervical dermatome/sensory level; T: thoracic dermatome/sensory level In laparoscopic cases, C3-C5 refers to sensory-only dermatomal coverage of the shoulder-tip region, aimed at preventing referred pain related to diaphragmatic irritation and pneumoperitoneum; no upper-limb motor block or respiratory impairment was observed

Case	ASA	Age	Procedure duration	Block onset	Max sensory level achieved	Analgesia duration / first rescue	Hemodynamic range	Respiratory range	Post-discharge follow-up
1	ASA II	29 years	50 min	10 min	C3-C5 sensory-only coverage for shoulder-tip pain; no upper-limb motor block	No rescue analgesia within 24 h	MAP 85-100 mmHg; HR 71-85 bpm; no vasopressor requirement	SpO₂ 98-99%; EtCO₂ 35-45 mmHg	POD 30 follow-up: no delayed neurological or respiratory complications.
2	ASA III	46 years	60 min	15 min	T8-T9	Analgesia duration 18 h; first rescue analgesia at 18 h	MAP 90-110 mmHg; HR 80-90 bpm; no vasopressor requirement	SpO₂ 98-99%; EtCO₂ 35-45 mmHg	POD 30 follow-up: no delayed neurological or respiratory complications.
3	ASA IV	78 years	65 min	10 min	C3-C5 sensory-only coverage for shoulder-tip pain; no upper-limb motor block	No rescue analgesia within 24 h	MAP 80-95 mmHg; HR 65-88 bpm; no vasopressor requirement	SpO₂ 94-95%; EtCO₂ 35-45 mmHg	POD 30 follow-up: no delayed neurological or respiratory complications.
4	ASA III	52 years	45 min	15 min	C3-C5 sensory-only coverage for shoulder-tip pain; no upper-limb motor block	No rescue analgesia within 24 h	MAP 85-100 mmHg; HR 68-90 bpm; no vasopressor requirement	SpO₂ 97-99%; EtCO₂ 35-45 mmHg	POD 30 follow-up: no delayed neurological or respiratory complications.

Case 1

**Figure 1:**
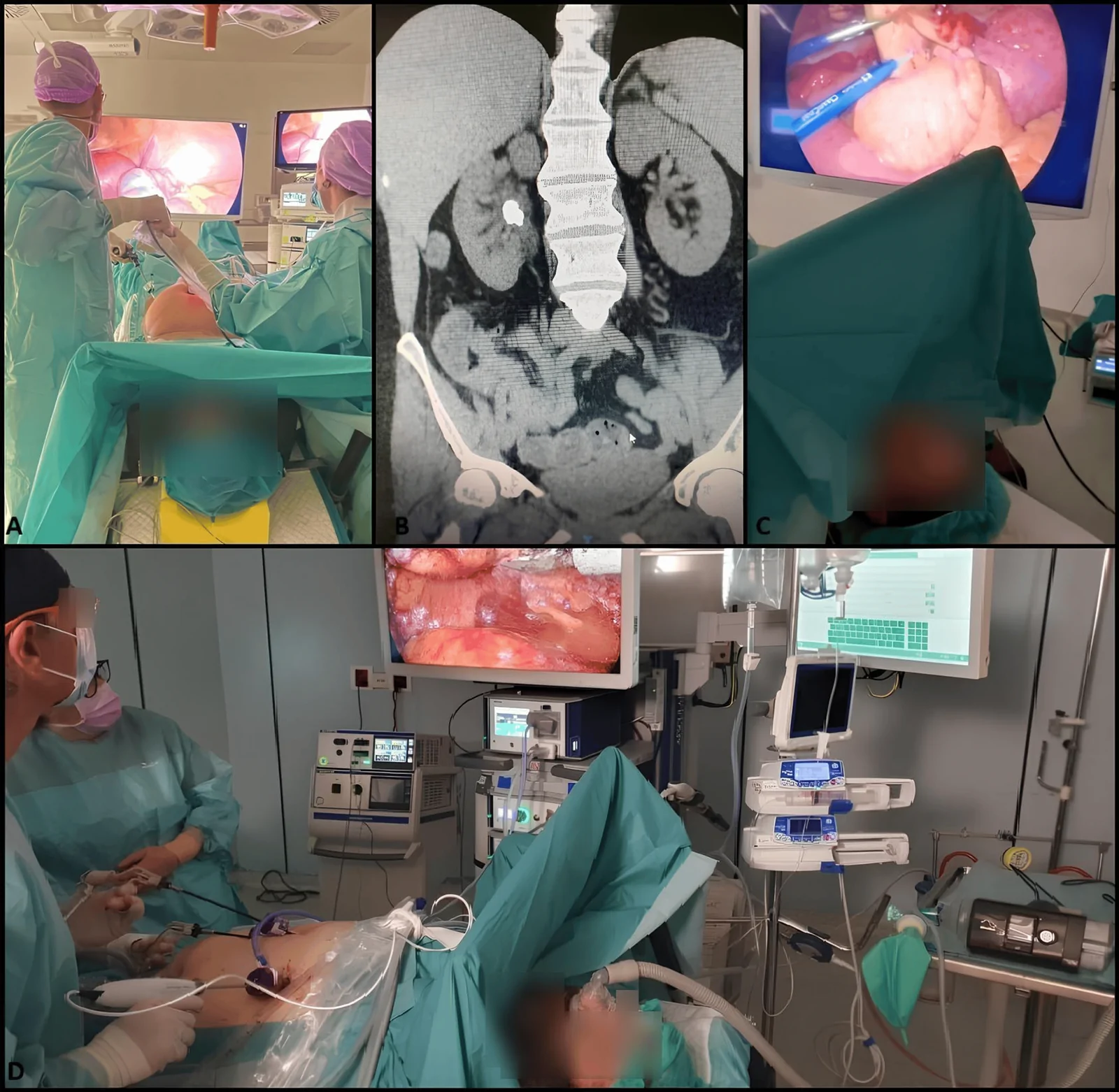
Representative image collage of the four cases managed with thoracic segmental spinal anesthesia (A) Case 1: Emergency videolaparoscopic adnexal detorsion in a first-trimester pregnant patient, performed under TSSA with preserved spontaneous breathing. (B) Case 2: Preoperative computed tomography image of right pelvic renal stones in a patient with arthrogryposis multiplex congenita and restrictive ventilatory impairment undergoing RIRS. (C) Case 3: Emergency laparoscopic appendectomy for acute gangrenous appendicitis in a patient with severe COPD, performed under TSSA while maintaining spontaneous breathing. (D) Case 4: Laparoscopic cholecystectomy in a morbidly obese patient with obstructive sleep apnea, performed under TSSA combined with CPAP support. TSSA: thoracic segmental spinal anesthesia; RIRS: retrograde intrarenal surgery; COPD: chronic obstructive pulmonary disease; CPAP: continuous positive airway pressure

A 29-year-old woman at eight weeks and six days of pregnancy, with class II obesity, was admitted for acute abdomen with suspected ovarian torsion. After multidisciplinary discussion, she underwent emergency videolaparoscopic adnexal detorsion with ovarian conservation (Figure 1A).

TSSA was performed at the T10-T11 level using sequential injection of hyperbaric bupivacaine 0.25% 5 mg and isobaric ropivacaine 0.25% 11.25 mg. The hyperbaric bupivacaine solution was freshly prepared by diluting commercially available 0.5% hyperbaric bupivacaine 1:1 with 0.9% sterile saline, obtaining a final concentration of 0.25% (5 mg in 2 mL). Mild intravenous propofol sedation was administered at 1 mg/kg/h while maintaining spontaneous breathing, with a target Richmond Agitation-Sedation Scale (RASS) score of -1 [9]. Pneumoperitoneum was well tolerated, without discomfort or respiratory compromise. Hemodynamic stability was excellent, with no vasopressor requirement. At the end of surgery, lower-limb motor recovery was complete, with a Bromage score of 0 [10]. Postoperative analgesia consisted of intravenous paracetamol, and the patient was discharged on postoperative day 2, reporting a favorable quality of recovery with a QoR-15 score of 142.

Case 2

A 46-year-old woman with arthrogryposis multiplex congenita, severe rotoscoliosis, and moderate-to-severe restrictive ventilatory impairment was referred for retrograde intrarenal surgery (RIRS) for right pelvic renal stones (Figure 1B). Because of the predicted respiratory risk and potential difficulty in airway management related to musculoskeletal deformities and reduced thoracic compliance, a segmental neuraxial approach was selected after collaborative preoperative evaluation.

TSSA was performed at the T11-T12 level using isobaric levobupivacaine 0.25% 10 mg plus dexmedetomidine 4 mcg as intrathecal adjuvant, followed by hypobaric levobupivacaine 0.25% 5 mg. The procedure was performed under spontaneous breathing, with a RASS score ranging from 0 to -1. Hemodynamic and respiratory conditions remained stable, with no vasopressor requirement. Pain control was excellent, and no neurological or respiratory complications were observed. At the end of surgery, lower-limb motor recovery was complete, with a Bromage score of 0. Postoperative analgesia included intravenous paracetamol and ketorolac. The patient was discharged on postoperative day 3 in good general condition, with a QoR-15 score of 144.

Case 3

A 78-year-old woman with severe chronic obstructive pulmonary disease (COPD), chronic cor pulmonale, and long-term oxygen therapy underwent emergency laparoscopic appendectomy for acute gangrenous appendicitis (Figure 1C). Given the severity of the underlying respiratory disease, avoidance of endotracheal intubation and controlled mechanical ventilation was considered potentially advantageous.

TSSA was performed at the T9-T10 level using sequential injection of hyperbaric bupivacaine 0.25% 5 mg (prepared by diluting 0.5% hyperbaric bupivacaine 1:1 with 0.9% saline, as described for Case 1) and isobaric ropivacaine 0.25% 11.25 mg, with intrathecal morphine 50 mcg as adjuvant. Low-dose intravenous propofol sedation was administered at 1 mg/kg/h, targeting a RASS score of -1. The patient remained easily arousable and tolerated pneumoperitoneum while maintaining spontaneous breathing with 3 L/min oxygen via nasal cannula. No vasopressor was required. At the end of surgery, the Bromage score was 0. No rescue analgesia was required postoperatively, and the patient was discharged on postoperative day 2. The QoR-15 score was 148.

Case 4

A 52-year-old man with morbid obesity, arterial hypertension, type 2 diabetes mellitus, and obstructive sleep apnea syndrome treated with home continuous positive airway pressure (CPAP) was scheduled for laparoscopic cholecystectomy for symptomatic cholelithiasis (Figure 1D).

TSSA was performed at the T11-T12 level using sequential injection of hypobaric ropivacaine 0.25% 5 mg followed by isobaric ropivacaine 0.25% 10 mg. Continuous dexmedetomidine infusion at 0.4 mcg/kg/h was used for sedation, targeting a RASS score of -1. The patient used his own home CPAP device intraoperatively, with a positive end-expiratory pressure of 8 cmH₂O. The procedure was completed uneventfully, without vasopressor requirement or respiratory deterioration. At the end of surgery, lower-limb motor recovery was complete, with a Bromage score of 0. No postoperative rescue analgesia was required, and the patient was discharged on postoperative day 2. The QoR-15 score was 145.

## Discussion

This four-case series highlights the potential role of TSSA as a modular anesthetic strategy in selected high-risk patients undergoing procedures from different surgical specialties.

Quantitative perioperative data are reported in Table 2. The mean age was 51.3 years (range, 29-78 years), the mean procedure duration was 55 minutes (range, 45-65 minutes), and the mean block onset time was 12.5 minutes (range, 10-15 minutes). ASA physical status ranged from II to IV. In laparoscopic cases, sensory-only cephalad spread included the C3-C5 shoulder-tip dermatomal region, aimed at covering referred pain related to pneumoperitoneum, without upper-limb motor block or respiratory impairment. QoR-15 scores were high in all patients, with a mean score of 144.8 and a range from 142 to 148. Intraoperative mean arterial pressure (MAP), heart rate, SpO₂, and EtCO₂ remained within clinically acceptable ranges in all cases. No patient required vasopressors, airway rescue, or conversion to GA. At 30-day post-discharge follow-up, no delayed neurological or respiratory complications were reported.

The central element linking these four cases was not the surgical procedure itself, but the presence of patient-specific factors that made preservation of spontaneous ventilation clinically desirable. These included first-trimester pregnancy with obesity, severe restrictive ventilatory impairment due to arthrogryposis multiplex congenita and rotoscoliosis, severe COPD with chronic cor pulmonale, and morbid obesity with obstructive sleep apnea requiring home CPAP. In these settings, GA remains feasible and may be necessary if regional anesthesia fails or surgical conditions require it. However, the risk-benefit balance may shift toward techniques capable of reducing avoidable respiratory and hemodynamic stress.

Patient selection and physiology preservation

TSSA should not be proposed as a routine substitute for GA. Rather, it should be considered when patient characteristics, surgical requirements, and anesthesiologist expertise converge. In our series, the anesthetic goal was consistent across different procedures: to obtain an adequate segmental block while maintaining spontaneous ventilation. This approach may be particularly attractive in patients with reduced respiratory reserve, predicted difficult airway management, or conditions in which positive-pressure ventilation and systemic anesthetic exposure could increase perioperative risk.

The concept of “physiology preservation” is central. Unlike GA with controlled ventilation, a successful TSSA-based strategy aims to preserve diaphragmatic function, spontaneous ventilation, airway reflexes, and early postoperative recovery, while providing sufficient sensory block for the surgical procedure. This does not imply that TSSA is intrinsically safer than GA in all high-risk patients. The favorable outcomes observed in this series likely reflect careful selection, appropriate case-by-case planning, and close collaboration with the surgical team.

Anatomical and technical considerations

A major concern with TSSA is the perceived risk of spinal cord injury during thoracic puncture. MRI studies have helped clarify thoracic spinal canal anatomy, showing that the spinal cord does not uniformly occupy the entire posterior subarachnoid space and that a measurable posterior cerebrospinal fluid space exists at thoracic levels [6]. These anatomical observations provide a rationale for cautious thoracic neuraxial techniques but should not be misinterpreted as eliminating risk. Technical precision remains essential.

Accordingly, TSSA should be performed only by clinicians familiar with thoracic neuraxial anatomy and low-dose intrathecal techniques. The use of small-gauge spinal needles, careful patient positioning, slow needle advancement, appropriate monitoring, and immediate availability of airway equipment and conversion to GA are mandatory elements of a safe strategy. The decision to perform TSSA should always include an explicit surgical and anesthetic backup plan.

Versatility across surgical specialties

Our series supports the concept that TSSA is not a niche technique limited to a single type of general surgery. It was used for emergency gynecological laparoscopy, endourology, emergency laparoscopic appendectomy, and laparoscopic cholecystectomy. This is consistent with the growing literature describing TSSA across multiple surgical domains [1-3].

Previous clinical studies have reported successful laparoscopic cholecystectomy under segmental thoracic spinal anesthesia [7,11]. However, the present series emphasizes a broader clinical message: the value of TSSA may lie less in a specific operation and more in its adaptability to different patients and surgical contexts. In this sense, the technique should be considered part of an anesthesiologist's armamentarium for selected high-risk cases rather than a fixed protocol for a single procedure.

Local anesthetic baricity and segmental spread

The use of local anesthetics with different baricity may allow more tailored distribution of the neuraxial block according to surgical requirements. In our cases, sequential injections of hyperbaric, isobaric, or hypobaric solutions were selected according to the clinical scenario. This was not intended as a rigid “double-baricity protocol,” but as a pragmatic way to influence cephalad and caudal spread while avoiding unnecessarily extensive sympathetic or motor blockade.

This may be particularly relevant during laparoscopic surgery, where anesthesia must address somatic surgical pain as well as visceral discomfort related to pneumoperitoneum and diaphragmatic irritation. Double-baricity techniques have been proposed to optimize segmental spread and reduce shoulder-tip pain during laparoscopic cholecystectomy under TSSA [12]. Previous opioid-free segmental thoracic spinal anesthesia experience has also reported cervical sensory spread, with sensory blockade extending to the C2-C3 region and a highly selective sensory involvement of cervical roots without impairment of diaphragmatic motor function [13]. In the laparoscopic cases of our series, sensory-only cephalad spread included the C3-C5 shoulder-tip dermatomal region, aimed at covering referred pain related to pneumoperitoneum, without upper-limb motor block or respiratory impairment. In our series, pneumoperitoneum was tolerated without clinically relevant discomfort or respiratory compromise.

Sedation and respiratory support

TSSA should not be equated with an “awake-only” technique. Sedation and respiratory support are not accessory elements, but integral components of a tailored anesthetic strategy. The aim is cooperative sedation: sufficient to improve comfort and tolerance of surgical stimulation, but not deep enough to impair spontaneous breathing, airway reflexes, or early postoperative recovery.

In this series, sedation was individualized. Low-dose intravenous propofol was used in Cases 1-3 to maintain cooperative sedation during spontaneous breathing, whereas continuous intravenous dexmedetomidine was used in Case 4 because of its sedative and anxiolytic properties with limited respiratory depression. Intrathecal adjuvants were used when prolongation or reinforcement of neuraxial anesthesia was desired. Previous reports have described the feasibility of opioid-free thoracic spinal anesthesia with intrathecal sedation for breast and axillary surgery, supporting the concept that sedation can be integrated into the neuraxial anesthetic plan [13].

Respiratory support was also individualized. Patients with preserved respiratory reserve maintained spontaneous breathing without ventilatory assistance, while CPAP was incorporated in the morbidly obese patient with obstructive sleep apnea. This reinforces the concept that the respiratory plan should be adapted to baseline respiratory physiology rather than standardized across all cases.

Relationship with previous reports

Previous reports by Scimia et al. described tailored TSSA in patients with advanced systemic sclerosis and Turner syndrome, supporting the concept that carefully individualized neuraxial anesthesia may represent a valuable option in selected high-risk patients in whom GA may carry additional hazards [14,15]. The present case series extends this concept by showing its application across multiple surgical specialties and different respiratory-support strategies.

Limitations

This report has several limitations. It describes a small, highly selected case series without a control group; therefore, no conclusions can be drawn regarding superiority over GA. The favorable outcomes observed may reflect careful patient selection, operator experience, and close collaboration with the surgical team. In addition, the heterogeneity of surgical procedures limits generalizability, although it also illustrates the potential versatility of the technique. Finally, the absence of complications in this small series should be interpreted cautiously, because rare neurological, hemodynamic, or respiratory adverse events cannot be assessed in such a limited sample.

## Conclusions

TSSA may represent a physiology-preserving anesthetic strategy capable of maintaining spontaneous breathing and reducing the perioperative burden of anesthesia in selected high-risk patients undergoing different types of surgery. Its value lies in careful patient selection, individualized intrathecal drug selection, appropriate modulation of local anesthetic baricity, titrated sedation, and integration of respiratory support when required. TSSA remains technically demanding and should be performed only by experienced practitioners, with appropriate monitoring, full airway equipment immediately available, and explicit agreement with the surgical team. Further prospective studies are warranted to better define the indications, safety profile, and short- and long-term outcomes of this technique in high-risk surgical patients.
